# App-based self-trainings targeting strain recovery and their effect on concentration

**DOI:** 10.1038/s41598-023-45906-6

**Published:** 2023-11-13

**Authors:** Martina Hartner-Tiefenthaler, Julia Schoellbauer

**Affiliations:** Institute for Management Science, TU Wien (Vienna University of Technology), Labor Science and Organization, Theresianumgasse 27, 1040 Vienna, Austria

**Keywords:** Human behaviour, Occupational health

## Abstract

During the COVID-19 pandemic, many knowledge workers reported concentration problems. This can be seen as critical as concentration is an important indicator for both cognitive wellbeing and occupational success. Drawing on the load theory of selective attention, we argue that concentration problems can be caused by the strain workers experienced during the pandemic. Consequently, by associating impaired concentration with strain, we hypothesize that strengthening strain recovery is a method that potentially supports concentration in stressful times. We developed the smartphone app "swoliba" containing self-training exercises targeting recovery experiences and tested the benefit of this app with two intervention groups and one waitlist-control group. Participants of the intervention groups were asked to carry out the exercises accompanied by surveys throughout a period of 4 weeks in 2020/2021. Results show that participants in the intervention groups reported higher concentration levels and lower strain levels than those in the control group, and this beneficial effect on concentration is partially mediated via lower strain levels. We conclude that self-training apps can be an effective tool for recovery interventions reducing strain but also supporting concentration. Using two different intervention conditions, we can reliably demonstrate the beneficial effect of our swoliba training program.

## Introduction

Concentration is an important indicator for both wellbeing and occupational success. It represents the absence of cognitive weariness and ensures that one can take up new information and make decisions^1^. Being able to concentrate on the task at hand is also a critical determinant for performance^2–4^, especially for workers with knowledge-intensive occupations. The majority of knowledge workers have to perform complex work tasks and solve unforeseen problems^5^, both of which require the highest level of cognitive regulation^6^ and thus concentration.

During the COVID-19 pandemic, knowledge workers reported to have concentration issues, which was reported by newspaper headlines such as “I have ‘pandemic brain’. Will I ever be able to concentrate again?”^7^ and similar others^8–10^. Boals and Banks^11^ pointed out that cognitive impairments during the pandemic were inevitable for even the most resilient individuals, and they argued that this is because the COVID-19 pandemic represented a considerably and unprecedentedly stressful time period for the majority of people. Drawing on this, we substantiate it theoretically using the load theory of selective attention and introduce the self-training app "swoliba" as practical, contemporary measure to support workers during stressful times.

Since the onset of the COVID-19 pandemic in 2020, workers were likely to be confronted with more stressors than in the years before^11^: Not only health stressors stemming from the virus per se, but also several other work-related stressors were likely. Most workers had little time to prepare for working from home which potentially decreased the predictability of workdays, and the unpredictability of a workday has been shown to be a work stressor^12^. Moreover, the threat of a recession as a consequence of the pandemic^13^ increased workers’ perceived risk to lose their job. Job insecurity is a fundamentally stressful work situation and affects the whole career^14^. As “high performance might be perceived as a safeguard against being laid off”^15^, job insecurity also indirectly puts additional demands on workers increasing their work efforts. Increased work efforts can either be expressed by longer working hours (i.e., doing more in more time) or by work intensification (i.e., doing more in the same time) which are both known for their negative association with occupational health^16^.

The pandemic also indirectly brought up work-related stressors of physical and social nature. Generally, physical stressors at the workplace are ambient, biochemical and posture-related risks and trigger a health impairment process indicated by exhaustion^17^. Due to COVID-19-induced restrictions, people were forced to keep a physical distance from colleagues and customers and primarily worked from home, a situation resulting in improvised workplaces (e.g., the kitchen table, the sofa, the kids’ desk). These ad-hoc adjustments were often not fully in line with ergonomic standards: Workspaces had too little or too much light, were noisy, or provided too little privacy to perform certain work tasks such as talking on the phone. The requirements of working and schooling from home made the appropriate work environment a scarce resource. Thus, a common social stressor was caused by the need to share scarce spatial resources with family members. Using private spaces for personal, occupational, and educational purposes, potentially causes conflicts between the family members but also between work and private life^18^. Work-life conflicts represent stressful situations that relate to lower levels of workers’ health and wellbeing as indicated for example by burnout, health problems, and depression^19^.

When a stressor needs to be overcome, but the individuals do not consider themselves fit to handle it, a stress response is triggered^20^. More specifically, a stress response—also referred to as strain—emerges when individuals expect that their reaction to the stressor will have no, an insufficient or a negative influence on the outcome of the situation^21^. In such situations, the sympathetic nervous system mobilizes the organism to act in response to the stressor^22^, leading to various psychophysiological reactions (i.e., high negative affective arousal^21^—e.g., feeling tense, alarmed, and upset^23^—as well as increased heart rate, blood pressure, muscle strength, mental activity, and total energy consumption) due to the release of catecholamines by the sympathetic nervous system^22^. Strained individuals are forced to invest compensatory effort to perform adequately (e.g., at work) which, in turn, amplifies their need for recovery even more^24^.

Recovery is defined as the process of returning the psychophysiological systems that were activated during strain to a baseline level^24^. When the stressor has passed, the parasympathetic nervous system keeps the organism in a calm and quiet state and aims at restoring the destructive effects of sympathetic arousal and restored energy that was consumed during the sympathetic activation^25^. Therefore, regular recovery from strain is important. In the light of continued exposure to stressors and incomplete recovery, stress responses can develop into “chronic health problems such as prolonged fatigue, chronic tension, persistent sleep problems, or manifest diseases”^25^. The recovery process is incomplete if the sympathetic systems remain activated and alerted by the stressor and cannot return to a baseline level^22^.

Particularly in stressful times, individuals need support to recover from strain because recovery experiences suffer when individuals face a high level of stressors^26,27^. The ability to recover from strain can be promoted by training^28,29^, and recovery trainings are interventions that usually aim at strengthening work-related recovery experiences as described by Sonnentag and Fritz^30^: the experience of psychological detachment from work stressors, of physical and mental relaxation, of mastery, and of control over one’s life. *Psychological detachment* from work refers to mentally switching off work and thus stopping to think about work outside working hours. *Relaxation* is characterized by a state of low activation and high positive affect. *Mastery* is defined as experiencing competence and proficiency and can be experienced by overcoming challenges. *Control* refers to individuals’ feelings of autonomy over the own life and the perception to be able to choose from at least two options^30^. There is empirical evidence that strain recovery interventions are successful ways to lower strain^28,31,32^, depression^33,34^, burnout, and anxiety^31^.

Recovery interventions often build on psychological detachment^33^. Our goal was to take a broader perspective and investigate all four recovery experiences^30^. Drawing on the effort-recovery model^24^, the recovery experiences trigger two complementary processes for recovery: First, psychological detachment and relaxation should bring diversion from work stressors and thus support the organism to cool down after a strain arousal. Second, experiencing mastery in and control over one’s life should build up psychological resources which strengthen individuals’ resilience and thus ability to cope with stressors^30^. To test the effect of these processes, we designed two intervention programs each encompassing both processes. Intervention 1 strengthened psychological detachment and control whereas intervention 2 strengthened relaxation and control (more information on the recovery experiences targeted by recovery interventions can be found in the method section). As we expected similar effects for both interventions, we state our first hypothesis addressing both interventions:

### Hypothesis 1

After a 4-week strain recovery intervention, workers of the intervention groups will report lower strain levels than workers of the control group.

After encountering a stressor, people not only experience feelings of strain, but also keep thinking about the stressor causing the strain^35,36^. In other words, strain evokes strain-induced cognitions that occupy cognitive resources that would be needed to focus one’s attention. Attention is the cognitive process that allocates an individual’s limited cognitive processing resources toward selectively chosen information associated with the current focus of attention^37^. Attention is “characterized by attempting to ignore some stimuli or aspects of stimuli defined as irrelevant, while concentrating on relevant processing”^38^. By drawing on the load theory of selective attention and cognitive control^37^, we argue that strain and its associated strain-induced cognitions produce perceptual load that occupy working memory resources because the working memory can only handle a certain load at any one time. Consequently, strain makes it hard for workers to think about anything else besides the stressor causing the strain.

Concentration is a cognitive function linked to the working memory and characterized by thought inhibition and selective focus of attention^39^. Empirical findings support the relationship between strain and concentration. Strain-related cognitions are associated with increased mind wandering^4,40,41^, reduced cognitive functioning^42^, and limited working memory capacity^43^ in experimental studies. Questionnaire studies show that workers’ ruminative thoughts about stressful work events^44^ and their need for recovery^2^ impair their concentration. Thus, we conclude in our second hypothesis:

### Hypothesis 2

Strain has a negative effect on concentration.

Strain seems to negatively relate to concentration and strain recovery might be a way to lower workers strain-induced perceptual load and thus to improve their concentration. Empirical findings about related interventions point in that direction. For example, a study with college students showed that participation in a stress-management intervention improved students’ working memory scores significantly as compared to a control group^43^. Moreover, Swanson et al.^45^ revealed that healthy sleep habits relate to high cognitive functioning. Moreover, park walks and relaxation exercises at lunchtime were found to improve concentration in the afternoon^46^. Consequently, we assume that recovery interventions might not only decrease workers’ strain, but also increase their concentration.

### Hypothesis 3

After a 4-week strain recovery intervention, workers of the intervention groups will report higher concentration levels than workers of the control group.

With regard to the underlying process for these effects, we draw on the load theory^37^ and propose: Strain induces thoughts about the stressor which occupy individuals’ focus of attention and, in turn, lower their concentration on the things they originally aimed to do (such as their work tasks). Strain recovery should, therefore, reverse this process and strengthen concentration in stressful times. Consequently, we argue that strain mediates the relationship between strain recovery and concentration leading to our fourth hypothesis.

### Hypothesis 4

Participation in a strain recovery intervention relates to higher concentration after 4 weeks, and this relationship is mediated via lower strain.

## Method

### Study design and procedure

In a mixed between–within subjects design, we had two intervention groups (IG 1 and IG 2) and one waitlist control group (CG). For the intervention, the strain recovery programs were administered electronically (e-intervention): Video, audio and text exercises were provided in German language via the smartphone app "swoliba" (= smart work-life-balance). Additionally, participants filled in questionnaires in 2-weeks intervals: at registration for the study (T0), 2 weeks (T1) and 4 weeks after the start of the intervention program or waiting period (T2). With a predicted medium effect size of *η*_*p*_^2^ = 0.08, an alpha level of 0.05, a desired power of 0.8, and three measures, the estimated total sample size was 111 (i.e., 37 participants per condition)^47^. Considering a drop-out rate of about 50% in the course of this study with multiple measurements, we aimed to recruit about 166 participants.

Participation in the study was limited to knowledge workers because they engage in complex work tasks during which concentration is especially needed^47^. Recruiting of participants took place between September 2020 and February 2021 by promoting the study as an opportunity to test a new smartphone app that supports recovery from strain and thus work-life-balance when working from home. To find participants during the COVID-19 pandemic, we posted study information in relevant social media groups, newsletters, placed online ads and distributed flyers in places in which knowledge workers could be found such as co-working places, cafés, fitness centers, or yoga studios. Moreover, we directly contacted work councils, consultants, and coaches and partnered with human resource managers in three organizations that sent an information about the study to their employees. To accelerate the recruiting progress, we offered monetary incentives (up to € 35 maximum) for study participation. In order not to risk biased clicking rates, incentives were only bound to completed questionnaires, but not to app usage.

Participation in the study was voluntary and started with filling out an online baseline questionnaire (T0) in which participants gave their informed consent for data usage. In the T0 questionnaire, participants were asked whether they wanted to start with the exercises immediately (and were thus assigned to the intervention groups) or 6 weeks later (and were thus assigned to the waitlist-control group). Participants from the intervention groups were randomly assigned to one of the two groups. Following this procedure, 89 participants enrolled until the end of 2020. Since intervention studies are likely to experience dropouts during the study period, we aimed for a higher initial sample. Excluding the Christmas time, we started a second recruitment phase mid-January 2021 and automatically assigned participants to one of the three groups ensuring that each group has an equal number of participants.

To access the online questionnaires, participants received links via e-mail and for IG1 and IG2 the links were also accessible in the app. After registering for the study, we called each participant individually and explained once more the procedure of the study as we depended highly on participants’ commitment. Additionally, we conducted bimonthly online Q&A sessions where we were available for any questions. We matched the questionnaires by assigning a unique number to each participant, which was automatically captured by clicking on the link to the survey. After the 6-weeks-waiting period, participants of the CG could also install the app and had access to the exercises of both groups.

### Ethical considerations

The study was conducted in accordance with the Helsinki Declaration (WMA, 2019) and complied with the highest ethical guidelines. Participants were informed about the goal and purpose of the study, and participation was completely voluntary. They could refrain from participation without any consequences at any time. Confidentiality was fully assured at any time. To organize the incentive payment, we had to ask for personal information, but this information was never included in the dataset. Also, the study did not involve individuals under the age of 18, did not deceive study participants, did not ask questions about intimate experiences or behaviors, did not include any drugs, placebos, or any other substances or potentially harmful procedures.

### Sample

Initially, 193 persons from Austria, Germany or Switzerland filled in the baseline-questionnaire (T0). Of those, 35 persons did not fulfill the recruitment criteria (i.e., legal age, having a knowledge-intensive job, and working at least 15 h per week) and were not invited to further participate in the study. Further 14 persons signed up for study participation but were not available for the obligatory briefing phone call or had decided to withdraw from participating. After data collection, we excluded 10 participants who did not use the app at all or only less than 8 clicks (i.e., viewed only less than 25% of the exercises) during a 4-weeks-period (albeit they were part of an intervention group). After the intervention, 29 participants were excluded due to the lack of filling in the questionnaire at T2 (after 4 weeks), which was necessary to test our hypotheses. Finally, three participants were excluded because they had experienced an above-average stressful life situation during the study (IG1: one participant lost the job, IG2: one participant suffered a serious injury, CG: one participant lost a family member).

The final sample consisted of 102 participants: 36 participants were assigned to IG1, 34 participants to IG2, and 32 participants to CG. We collected data at T0 and T2 (after 4 weeks) from all 102 participants, 95 participants additionally filled in the T1 questionnaire (after 2 weeks). Most participants in each group were female (IG1: 72.2%, IG2: 67.6%, and CG: 65.6%) and participants’ average age was 40.2 years (*M*_*IG1*_ = 39.8 years, *SD*_*IG1*_ = 12.0; *M*_*IG2*_ = 41.9 years, *SD*_*IG2*_ = 11.6; and *M*_*CG*_ = 38.8 years, *SD*_*CG*_ = 12.2). The majority lived with their partner in one household (IG1: 61.1%, IG2: 61.8%, and CG: 62.5%) having on average 0.4 kids (*M*_*IG1*_ = 0.6 kids, *SD*_*IG1*_ = 0.9; *M*_*IG2*_ = 0.3 kids, *SD*_*IG2*_ = 0.6; and *M*_*CG*_ = 0.3 kids, *SD*_*CG*_ = 0.7). Across all groups, participants were employed for 34.6 h per week (*M*_*IG1*_ = 34.1 h, *SD*_*IG1*_ = 7.5; *M*_*IG2*_ = 34.7 h, *SD*_*IG2*_ = 6.1; and *M*_*CG*_ = 35.1 years, *SD*_*CG*_ = 7.4). To check for potential attrition bias, we compared the T0 means and socio-demographics between the final sample (n = 102) and the dropouts (n = 56), but did not find any significant differences regarding gender, age, education, job tenure, working hours, partnership, kids living in the household, strain, and concentration.

## Intervention

Our self-training recovery intervention program was based on Sonnentag and Fritz’^30^ widely resonated theory of recovery introducing the four recovery experiences psychological detachment, relaxation, mastery, and control. In order to test the robustness of the effect of recovery interventions on strain and concentration, we designed two intervention programs. Both programs were supporting immediate recovery and strengthening resilience based on 33 exercises encompassing 7 video, 7 audio and 19 text instructions.

### Intervention 1: Strengthening psychological detachment and control.

Intervention program 1 contained exercises that aimed to help workers to actively manage their boundary between their work and their private life roles such as fostering psychological detachment from work and building up control experiences. Table 1 gives an overview and examples of the exercises.

**Table 1:** Example exercises of the recovery intervention 1 (Strengthening psychological detachment and control).

Mechanism to strengthen psychological detachment and control	Number of exercises	Example exercise
Distraction and disengagement from work-related thoughts	10	"Unload your thoughts": Your mind is still buzzing with work after work? Then consciously exchange your thoughts with another person. Start with your negative experiences (What didn't work out for you today? What stressed you out today?) and end with positive experiences (Did you experience success today? What situations at work made you laugh?) so you can start your leisure time with a nice thought. Exchange work experiences with one another but, after that, close the topic of work, because there are so many other interesting topics to discuss!
Psychological anchors	3	"Leisure anchor": Symbols surround us always and everywhere and we usually associate them with certain information. For example, with a red traffic light you probably associate "Attention, stop!", the smell of cinnamon probably reminds you of Christmas, and hearing the sound of the sea probably transfers you into a vacation mood. Is there also something you associate with your leisure time that and would simultaneously never associate with work? In this video, we'll show you a few examples of how places, people, objects, or smells can serve as leisure anchors for you
Transition rituals	15	"Changing robe": You've finished your workday at the home office? Then it's time for leisure! Signal this to your head as well by performing a small action to clarify to your mind the demarcation between work and leisure: (1) When taking off your work clothes, imagine that you are literally brushing off your work. (2) Then slip into your leisure clothes and consciously think about what you want to do first in your leisure time
Boundary rules	5	"Accessibility Rules”: Without interruptions we are more productive. We can concentrate better on our current work activities, but we can also make better use of our leisure time without unexpected interruptions, be it for our relaxation or to be able to enjoy a barbecue with the family. Therefore, (1) formulate rules about when and where you don't want to be available for anyone. (2) Think about what steps you need to take to stick to your own rules (e.g., activate the voice box, write availability times in the messenger profile information). (3) Think about whom you want to inform about your rules. (4) Consistently put your phone on silent or airplane mode at times when you don't want to communicate with anyone and put it in a place out of your sight

*Psychological detachment* from work is impaired when workers experience a discrepancy between their expectations of something (i.e., their goal) compared to the actual situation. This discrepancy can either be negative (i.e., the workday was less successful than expected) or positive (i.e., the workday was more successful than expected). To stop such intrusive thoughts you can distract yourself or disengage from the goal^36^. Thus, our exercises supported distraction and disengagement from work during leisure time. Drawing from work-life segmentation literature (see e.g.,^48–50^), we further designed exercises to establish psychological anchors from leisure time and to define transition rituals. The goal was to help participants to more easily distance from their work (issues) after working hours.

*Control* refers to individuals' feelings of autonomy. Connected to the context of the study, we primarily focused on boundary control asking workers to establish rules associated with their life roles and their boundaries or by planning the day ahead to make the day more predictable^12^. Defining rules and planning help to make them aware that they have the ability to choose their actions from two or more options which determines their experience of control^30^.

### Intervention 2: strengthening relaxation and mastery

Intervention program 2 aimed at strengthening relaxation and fostering mastery experiences. This program primarily focusses on activities during leisure time before or after work or during breaks on work days. Table 2 provides examples of the exercises used.

**Table 2:** Example exercises of the recovery intervention 2 (Strengthening relaxation and mastery).

Mechanism to strengthen relaxation and mastery	Number of exercises	Example exercise
Relaxation	12	"Vacation in thought": How often are we longing for our well-deserved vacation. But really, we don't need a plane ticket to feel lighter and a hammock to unwind for a moment. With a little practice, if we close our eyes, we can get to our very own place of rest and relaxation. Give yourself a time out; we'll guide you to it
Sleep	5	“Bedtime story”: Use this exercise to remind yourself to read a few more pages before bed. Maybe you have a good book you've been meaning to read, or have a bedtime story read to you as an audiobook. This works for kids, so why not for adults? We've recorded a few short stories for you. Feel free to listen in, or listen/read your own book
Good mood	10	"Rest reminder": In our high-performance society, it sometimes happens that we neglect our rest and (feel like we) are working all the time. Therefore, with this exercise, remind yourself to take a break. After a regular rest you will be refreshed and thus be able to tackle your tasks with new energy!
Self-efficacy beliefs	6	"Focus on strengths": When you ask people about their strengths, you usually get rather hesitant answers. We often know more about our failures. You can change that with this exercise! Look back on your day and become aware of your strengths that you were able to apply today: (1) Become aware of your strengths. (2) Review your day and identify situations where you were able to use your strengths today (e.g., Which small or big things did you master today? Which strengths can you derive from this for yourself?)

*Relaxation* defines a state of low activation and high positive affect. To foster relaxation, we created exercises that helped workers to relax during their leisure time such as exercises promoting calmness (by going into the nature) or muscle relaxation. Since relaxation is associated with a good night’s sleep^51^, we also aimed to improve participants’ sleep quality. Sleep disturbances are associated with the pre-sleep psychological state^52^. Thus, we particularly targeted the time before going to sleep.

*Mastery* refers to a feeling of accomplishment. The program contains activities offering learning opportunities without exceeding individuals’ capabilities^30^. As mastery experiences are associated with positive mood^53^ and self-efficacy^54^, our exercises aimed to improve positive mood and support experiencing self-efficacy.

### Developing and pre-testing the smartphone app "swoliba"

Both interventions were administered via the smartphone app "swoliba". In collaboration with the research group INSO (industrial software) from the Informatics Department at TU Wien, the app was developed in an iterative and lengthy process particularly for this study. It was compatible with iOS and android smartphones and available via the Apple app store and the Google play store. After installing the app and actively giving informed consent to the study, participants received instructions about how to use the app via a video manual that started automatically. We informed them about the app’s aim to strengthen strain recovery and thus “work-life-balance” and clarified that the success highly depends on their regular exercise over the period of at least 4 weeks.

To increase usability, we designed exercises of varying length and declared them as “1-min-exercises”, “3-min-exercises”, and “5plus-min-exercises”. Also, the exercises were tagged as either specifically applicable in the morning (i.e., before or shortly after the beginning of a workday), in the evening (i.e., shortly before or after the end of a workday), or generally at any time of the day the participant chooses. This resulted in three to four exercises per category (duration * time of day). After selecting an exercise, participants were asked to define a concrete time when they want to carry it out (from next Monday to Sunday). Based on the scheduled time for each exercise, the app contained an alert function reminding the participants to perform the exercise. Additionally, a week planner provided them with an overview of the selected exercises and the possibility to mark the exercises as completed.

Before the final release of the app, we had three consecutive pilot studies. The first pilot study was conducted among colleagues and friends of the project team. We collected feedback with the method of thinking aloud^55^ and refined the exercises and the functionality of the app. For the second pilot study, we recruited a sample of workers who used the app for several weeks. Their feedback was used to further improve the functionality of the app. After these improvements, we conducted a third and final pilot study with students. As part of course credits, they had to write a reflection about their most favorite exercises. Based on this feedback, we further adjusted the exercises to improve their attractivity.

## Measures

The three questionnaires (T0–T2) were completed online. All study variables were assessed at all three measurement points, except questions about socio-demographics that were only asked once. Participants were asked to answer all items on a 7-points Likert scale ranging from 1 (= never) to 7 (= all the time). The respective item responses were averaged to build the strain and concentration scale.

*Strain* was assessed with four items based on Warr et al.^23^ asking participants to indicate to what intensity they felt “tense”, “alarmed”, “upset”, and “uneasy” in the last week during their leisure time. Cronbach’s alpha of the scale ranged between 0.74 (T1) and 0.86 (T2).

*Concentration* was assessed by three items based on the subscale concentration of Sneddon et al.’s^4^ situational awareness scale. The items were formulated reversed, so participants were asked to indicate to what extent they lacked concentration throughout their last work week. A sample item reads “Last week, I was not able to keep my mind focused and it had a tendency to ‘wander’”. We recoded the scale to picture workers’ level of concentration (i.e., the higher the more concentration). Cronbach’s alpha of the 3-item scale ranged between 0.79 (T1) and 0.88 (T0).

*Unusual events* during the study period were identified by asking “Did something out of the ordinary happen in your life this week that kept you very busy, whether it was positive or negative, personal or professional?”. Based on this question, data from three participants were excluded (see sample description).

### Statistical analysis

IBM SPSS Statistics 26 and Mplus Version 8^56^ were used for analyzing the data. First, we conducted a multivariate analysis of variance (MANOVA) to test whether socio-demographics and the baseline scores of the study variables differed between IG1, IG2, and the CG. Subsequently, we tested the main effect of the group on T2 strain and T2 concentration while controlling for the strain and concentration levels at T0 and T1 (Hypotheses 1 and 3). We opted for MANOVA as the two dependent variables (i.e., strain and concentration) are not independent from each other. We also reported univariate ANOVA results and simple contrasts. To inspect time effects, we complemented the analysis with mixed ANOVAs.

**Figure 1:**
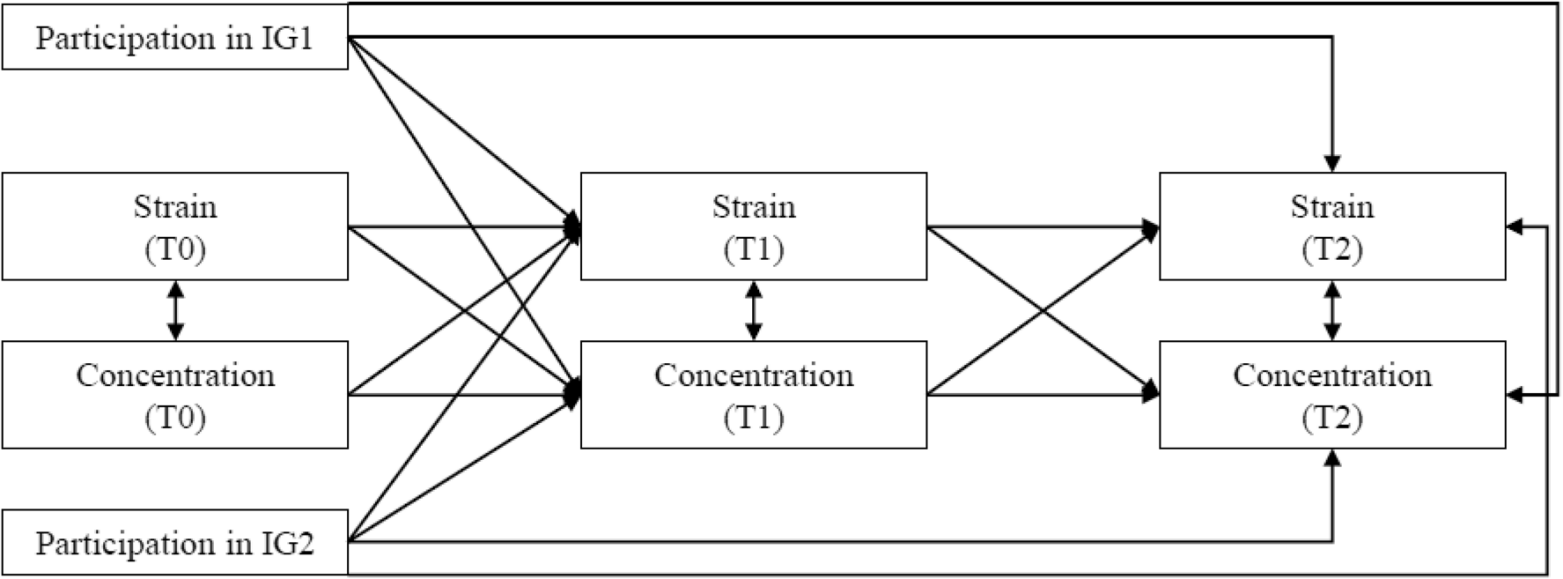
Longitudinal mediation path model. One-sided arrows symbolize modeled paths in a certain direction, two-sided arrows symbolize two-tailed correlations.

Finally, to test Hypotheses 2 and 4, we conducted a multivariate regression analysis using path modelling in Mplus. We modelled strain and concentration at all three measurement points into the model and used dummy coding for testing the effect of IG1 and IG2. We first modelled the paths from the IG1 and IG2 dummy variables to strain at T1 and to concentration at T2 and from strain at T1 to concentration at T2 because these paths represent what was proposed in Hypothesis 2 and 4. Then we entered all other possible paths between the variables (i.e., cross-lagged paths) in order to derive information about the causality of their relationship (see Fig. 1). We also allowed all variables collected at the same measurement time to correlate. The model showed a good model-fit, χ^2^ (8) = 31.6, comparative fit index (CFI) = 0.89, standardized root mean square residual (SRMR) = 0.07, and Akaike Information Criterion (AIC) = 1662.3. Finally, 95% confidence intervals (CI) were calculated by applying the Monte Carlo method^57^ with 1,000,000 repetitions.

### Manipulation check

As the app counted each click on each exercise per participant, we could check for manipulation based on objective electronic data. Checking the usage of the app is essential as the sole installation of the app will not have a beneficial effect and regular usage is crucial for the success of the intervention. Throughout the 4 weeks of intervention, participants clicked 6896 times on any of the 66 exercises, yielding an average of 104 clicks per exercise and 99 clicks per participant. In both intervention groups participants dealt on average with 14 exercises per person. In intervention group 1, participants used 3 to 31 exercises whereas in intervention group 2, they used 2 to 33 exercises.

## Results

Table 3 contains the means and standard deviations of strain and concentration at all measurement points differentiating between IG1, IG2, and the CG. Zero-order correlations and Cronbach’s alphas for all study variables are shown in Table 4.

**Table 3:** Means and standard deviations of the study variables.

Variable	Group	T0		T1		T2	
		*M*	*SD*	*M*	*SD*	*M*	*SD*
Strain	IG1	3.43	1.12	3.18	0.98	3.05	1.14
	IG2	3.43	1.31	3.11	1.25	3.02	1.20
	CG	3.66	1.17	3.87	0.97	3.63	1.22
Concentration	IG1	4.47	1.26	4.73	1.07	4.87	1.22
	IG2	4.91	1.15	4.88	1.02	5.07	1.17
	CG	4.48	1.35	4.23	1.20	4.18	1.12

IG1 = intervention group 1, IG2 = intervention group 2, CG = control group; T0 = baseline; T1 = after two weeks of exercising (during intervention), T2 = after four weeks of exercising (after intervention), n_IG1_ = 35 (T0), 34 (T1), 36 (T2); n_IG2_ = 34 (T0), 33 (T1), 34 (T2); n_CG_ = 32 (T0), 28 (T1), 32 (T2).

**Table 4:** Zero-order correlations and reliability of the study variables.

	1	2	3	4	5	6	7	8	9
1 Participation in IG1									
2 Participation in IG2	− 0.52**								
3 Participation in CG	− 0.50**	− 0.48**							
4 Strain T0	− 0.05	− 0.04	0.09	(0.83)					
5 Strain T1	− 0.12	− 0.16	0.30**	0.68**	(0.74)				
6 Strain T2	− 0.11	− 0.12	0.23*	0.57**	0.65**	(0.86)			
7 Concentration T0	− 0.09	0.17	− 0.08	− 0.56**	− 0.43**	− 0.36**	(0.88)		
8 Concentration T1	0.06	0.16	− 0.24*	− 0.43**	− 0.56**	− 0.36**	0.55**	(0.79)	
9 Concentration T2	0.09	0.20*	− 0.30**	− 0.45**	− 0.56**	− 0.57**	0.62**	0.56**	(0.84)

Participation in IG1 = participants of intervention 1 were coded as 1 and all other participants as 0 (i.e., participants of IG2 and CG), participation in IG2 = participants of intervention 2 were coded as 1 and all other participants as 0, participation in CG = participants of the control group were coded as 1 and all other participants as 0. *p = 0.05. **p = 0.0.

### Preliminary analyses

Before testing the hypotheses, we tested for differences between IG1, IG2, and the CG. Using Wilks’s statistic, the MANOVA yielded no significant differences of socio-demographics and baseline scores between the three groups, *Λ* = 0.88, *F*(14, 170) = 0.79, *p* = 0.68. Group differences of the following variables were tested: gender (*F*(2, 91) = 0.00, *p* = 0.996), age (*F*(2, 91) = 0.79, *p* = 0.46), partnership (*F*(2, 91) = 0.20, *p* = 0.822), children living with them in one household (*F*(2, 91) = 1.39, *p* = 0.26), working hours per week (*F*(2, 91) = 0.23, *p* = 0.79), baseline scores of strain (*F*(2, 91) = 0.18, *p* = 0.84) and also baseline scores of concentration (*F*(2, 91) = 1.19, *p* = 0.31).

### Hypotheses test

Analyzing group differences between strain and concentration at three points in time (T0, T1 and T2) using MANOVA, there was a significant effect of the recovery intervention on strain and concentration, *Λ* = 0.75, *F*(12, 172) = 2.24, *p* < 0.05, *η*_*p*_^2^ = 0.14. Hypothesis 1 proposes that participants of the intervention groups would report lower strain levels as compared to those of the control group which did not engage in a recovery intervention over a course of 4 weeks. In line with this hypothesis, the univariate ANOVA revealed differences of strain at T2, *F*(2, 91) = 3.86, *p* < 0.05, *η*_*p*_^2^ = 0.08. This effect was already prevalent at T1, *F*(2, 91) = 5.19, *p* < 0.05, *η*_*p*_^2^ = 0.10. Simple contrast analysis shows in line with our assumption that strain is lower in IG1 (T1: ∆ = − 0.77, *p* < 0.05, T2: ∆ = − 0.78, *p* < 0.05) and IG2 (T1: ∆ = − 0.76, *p* < 0.05, T2: ∆ = − 0.71, *p* < 0.05) compared to the CG.

**Figure 2:**
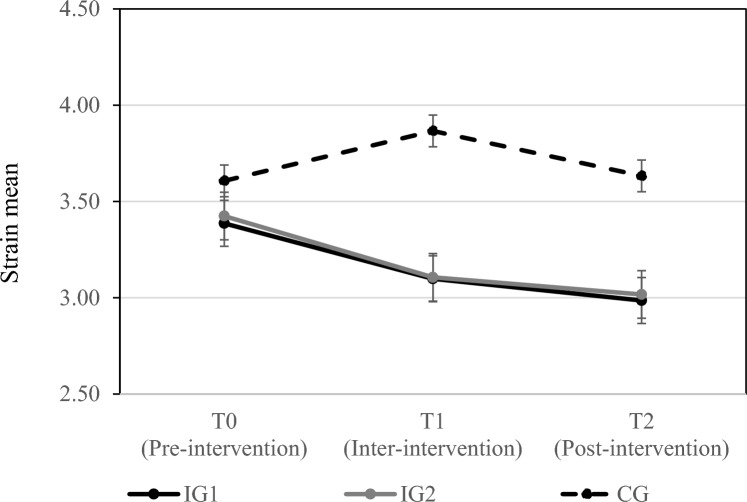
Development of means of strain (scale ranged between 1 and 7) for the three study groups and before (T0), during (T1), and after the four intervention weeks (T2). The error bars show the standard errors.

We inspected time effects via mixed ANOVA and obtained significant interaction effects for time and groups using Wilks’s statistics *Λ* = 0.84, *F*(8, 176) = 2.06, *p* < 0.05, *η*_*p*_^2^ = 0.09. Univariate results showed that for strain there was no statistically significant interaction between the intervention and time, *F*(4,182) = 2.01, *p* = 0.10, η_p_^2^ = 0.04. Thus, we found no variation of the effect over time on strain (see Fig. 2 for means over time).

Hypothesis 2 postulates a negative effect of strain on concentration and was tested in the multivariate regression analysis. As displayed in Table 5, our longitudinal path model yielded a negative relationship between strain at T1 and concentration at T2 whereas no relationship between concentration at T1 and strain at T2. These results support H2 and thus provide evidence that lower strain increases concentration.

**Table 5:** Results from the longitudinal path model.

	Strain T1	Concentration T1	Strain T2	Concentration T2
Participation in IG1	− 0.25** (0.08)	0.23* (0.11)	− 0.06 (0.11)	0.10 (0.09)
Participation in IG2	− 0.25** (0.08)	0.20^†^ (0.10)	− 0.04 (0.10)	0.15 (0.08)
Strain T0	0.65*** (0.09)	− 0.17 (0.11)		
Concentration T0	− 0.07 (0.08)	0.47*** (0.10)		
Concentration T1			0.02 (0.08)	0.36*** (0.10)
Strain T1			0.64*** (0.08)	− 0.32** (0.11)

Standardized estimates and standard errors (in parentheses) are reported.

***p > 0.001, **p < 0.01, *p < 0.05, ^†^*p* <0 .10..

Hypothesis 3 states that participants of the intervention groups would report higher concentration levels as compared to those of the CG after 4 weeks of intervention. Again, the univariate ANOVA supports the hypothesis and shows differences of concentration at T2, *F*(2, 91) = 5.43, *p* < 0.05, *η*_*p*_^2^ = 0.11 and the effect was already observed at T1, *F*(2, 91) = 3.28, *p* < 0.05, *η*_*p*_^2^ = 0.07. Simple contrast analysis shows in line with our assumption that concentration is lower in IG1 (T1: ∆ = 0.57, *p* < 0.05, T2: ∆ = 0.79, *p* < 0.05) and IG2 (T1: ∆ = 0.65, *p* < 0.05, T2: ∆ = 0.95, *p* < 0.05) compared to the CG.

**Figure 3:**
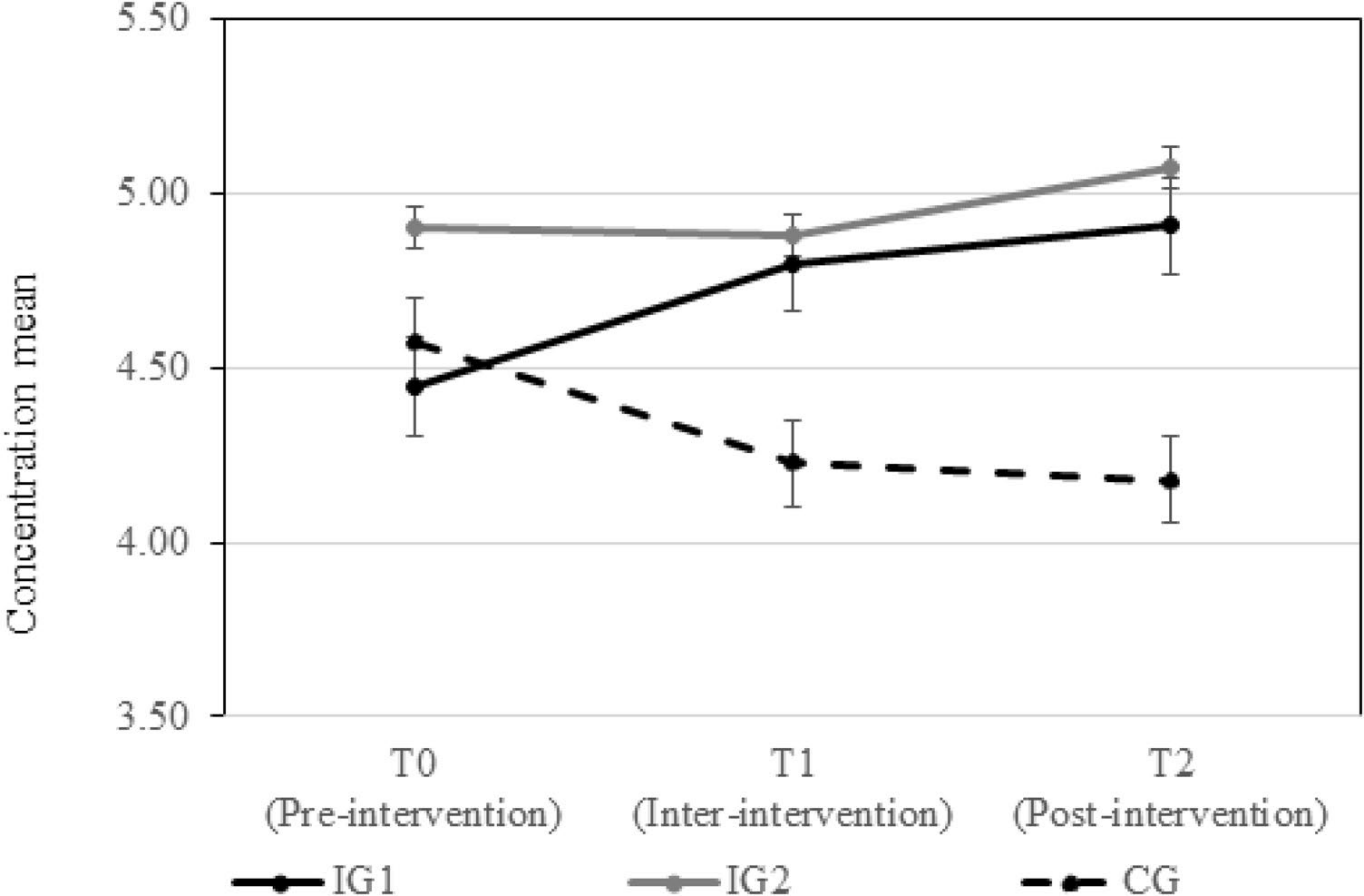
Development of means of concentration (scale ranged between 1 and 7) for the three study groups and before (T0), during (T1), and after the four intervention weeks (T2). The error bars show the standard errors.

Univariate result from the mixed ANOVA revealed a significant interaction between the intervention and time on concentration, *F*(4,182) = 2.01, *p* = 0.10, η_p_^2^ = 0.04. Thus, this result indicates that the group effect on concentration depends on time passing by in the course of the study. Figure 3 shows that the increase of concentration is immediate and steep for IG1 (∆_2–1_ = 0.34, ∆_3–2_ = 0.13) whereas for IG2 (stay positive) it seems that the effect only evolves after T2 (∆_2–1_ = − 0.02, ∆_3–2_ = 0.21).

Hypothesis 4 proposes that participating in the intervention indirectly predicts higher levels of concentration via lower levels of strain. Our results support this hypothesis and show a significant negative effect of IG1 and IG2 on strain at T1. Strain at T1, in turn, significantly predicted lower levels of concentration at T2. Consequently, we found an indirect positive effect from IG1 (*β* = 0.08, *SE* = 0.04, CI 95% [0.016; 0.163]) and IG2 (*β* = 0.08, *SE* = 0.04, CI 95% [0.016; 0.196]) on concentration at T2 mediated via lower strain levels at T1. The standardized regression estimates are displayed in Table 5.

To test for the influence of the control variables, we also calculated a mediation without any control variables, as described by Jose^58^. Our results remained stable as the direct effects between IG1 and strain at T1 (*β* = − 0.27, *SE* = 0.11, *p* < 0.01), between IG2 and strain at T1 (*β* = − 0.31, *SE* = 0.12, *p* < 0.01) as well as between strain at T1 and concentration at T2 (*β* = − 0.51, *SE* = 0.09, *p* < 0.001) were significant in the reported direction.

## Discussion

Concentration is an important indicator for workers’ cognitive wellbeing^1^ and a critical determinant for performance^2–4^. It is, however, compromised when individuals are exposed to high levels of stress, which clearly was the case during the COVID-19 pandemic^11^. Drawing on the load theory of selective attention^37^, we assumed that strain triggers thoughts and emotions occupying attentional resources and thus lowering workers’ concentration on (work) tasks. Using two app-based self-training programs aiming at strengthening different recovery experiences (as described by Sonnentag and Fritz^30^), we investigated how strain reduction increases concentration. More concretely, we tested whether recovery interventions, which were shown to have beneficial effects on strain^28,31^, also have beneficial effects on concentration via lower strain levels.

Our results show that app-based recovery self-trainings have a robust effect on strain reduction and on strengthening concentration. After 4 weeks of participating in a self-training program supporting their strain recovery, workers of the intervention groups reported significantly lower levels of strain and higher levels of concentration as compared to workers of the control group. Our findings on a reduction of strain is in line with prior group recovery interventions^28,31,32^ and mood-enhancing self-training interventions^59,60^ showing reductions in participants’ strain levels after the intervention. However, we are the first to show a beneficial effect of recovery interventions on workers’ concentration and thus add another benefit to recovery trainings. Using two intervention groups, we underpin the robustness of the beneficial effect of recovery interventions that both support workers to cool down after strain arousal and support them building psychological resources that protect them from experiencing strain. Although both intervention programs were effective, it seems that IG2 (strengthening relaxation and mastery) needs more time than IG1 (strengthening psychological detachment and control) for unfolding the beneficial effect on concentration.

Furthermore, the longitudinal study design allowed us to test the causal effect of strain on concentration. We tested all possible mediation paths in our longitudinal mediation analysis. Higher levels of concentration are likely after 4 weeks of the strain recovery intervention program, which is mediated via higher concentration levels but also via lower strain levels after 2 weeks. Furthermore, as concentration at T1 does not predict later levels of strain (T2), but strain at T1 predicts later levels of concentration (T2), findings of our longitudinal analysis support the causality of the effect of strain on concentration—probably for the first time—outside of experimental settings^43,61^.

Using a smartphone app for our strain recovery intervention might have enhanced participants’ motivation and engagement compared to traditional face-to-face interventions since it allows participants to autonomously decide which, when and where participants want to conduct their exercises. Feeling autonomous and in control is a basic human need and crucial to develop autonomous motivation which is associated with personal growth and wellbeing^62^. Participants’ feelings of autonomy can further be increased by supporting their self-leadership^63^ which was realized by integrating a personal exercise calendar in the app. Planning the day or week encompasses the setting of specific goals the person aims to accomplish, which leads to higher performance than “vague, abstract goals such as the exhortation to ‘do one’s best.’”^64^. Moreover, an app allows the integration of gamification elements (e.g., collecting points, progress bars) and a variety of ways how the content is displayed such as text, images, audio or video with the possibility to link to further information (e.g., we included a link with the theoretical background of each recovery exercise). Gamification elements are also known to increase motivation to stick to the program^65,66^. Rich information produce a high perceptual load in the working memory which makes it more likely that the training exercises are the sole focus of attention and the participant does not get distracted from doing them^67^.

Regarding advantages of app-based self-trainings for the research process, we argue that they provide technological features that allow the collection of objective information on whether participants have participated in the training (e.g., by counting the clicks in the app or the duration they actively dealt with the study app). “Manipulation checks are a valuable means of assessing the robustness of experimental results in studies based on subjects’ attention to treatments”^67^ and are thus crucial for intervention studies. An app can further provide a pseudonymous way for communicating between researchers and study participants. If a chat or messaging function is integrated in the app, the study-related communication can take place independently of a personal email address or telephone number and does not allow any conclusions to be drawn about a particular natural person. Using an app further enables the researcher to send timely reminders for carrying out the exercises which compensated for human obliviousness.

Our study contributes to the literature in three ways. First, we provide empirical evidence showing that strain reduction increases workers’ concentration. Based on our a strong research design, we could test for causal effects due to the longitudinal nature of our data^58^ illuminating the mechanism by which the intervention has an impact on concentration^68^. Also, by designing and testing two intervention conditions via a smartphone app targeting different recovery experiences, we demonstrated the reliability of the effects of our training program on strain and concentration.

Second, by using a smartphone app to administer the self-training, we tested a contemporary tool that holds many benefits for the conduction of recovery interventions for knowledge workers as compared to traditional intervention methods. In the past, recovery interventions were conducted in group settings with a trainer^28,31,69–71^. However, app-based support programs have been highlighted as new and more accessible ways to reduce stress^72^ and support mental health, such as in the treatment of bipolar disorder^73^. We thus argue that conducting an intervention targeting strain recovery via an app-based self-training is not only an appropriate reaction to the pandemic-induced change towards physical distancing and e-learning, but also holds advantages regarding the external validity of the intervention and participants’ motivation to participate in the program.

Third, we provide a theoretical ground to the concentration (or mind-wandering) literature which has been described as “long on results and short on theory”^74^. Drawing on the load theory of selective attention^37^, we explain that strain induces thoughts about the stressor which occupy scarce attentional resources and, in turn, lower workers’ focus on other, not stressor-related tasks. We could show that strain recovery reverses this process and strengthens concentration. This underlying rationale might also be applicable for other outcomes than concentration. As Diamond^39^ described focused attention as one of several functions relevant for reasoning, problem-solving, and planning, it would be indicated to investigate the implications of strain recovery on other functions such as working memory (e.g., mental math, relating one fact to another), cognitive flexibility (e.g., being able to see something from many different perspectives, to quickly switch between tasks), and the ability to exert self-control and discipline.

Despite its merits, our study is not without limitations. First, although we randomly grouped participants to both intervention conditions, we initially let participants choose their preferred date of start that defined intervention groups vs. control groups. However, as the three study groups did not show significant differences regarding sociodemographic characteristics as well as strain and concentration levels at the beginning of the study, we do not believe that our approach made our findings less valid. Second, although we were able to objectively check if the participants clicked in the app, unfortunately our app did not provide many details. We only had the sum of exercises clicked over the course of 4 weeks. In order to deduce information on the intensity of their app usage over the course of the study, it would have been necessary to know how many exercises the participants viewed on each day or at least per week.

Future research should not only overcome our study’s limitation but should further explore time effects and the potential of strain recovery on cognitive functions. Also, it would be interesting to investigate further potential mechanisms in which recovery interventions affect cognitive functions. Our longitudinal analysis reveals a positive indirect effect of recovery intervention on concentration via lower strain, but it also shows a significant direct effect of participation in a strain recovery intervention on concentration. From this finding it can be deduced that strain reduction may not be the only way in which strain recovery affects concentration. Nonetheless, our study shows that strain recovery is a way to conquer concentration issues. Promoting concentration is becoming more and more relevant in our modern life due to potential distractions and interruptions and also represents a mental health function that is associated with performance^2–4^ and wellbeing^1^.

## Data availability

Data and material are available on request from the corresponding author.
